# Identification of a genomic DNA sequence that quantitatively modulates KLF1 transcription factor expression in differentiating human hematopoietic cells

**DOI:** 10.1038/s41598-023-34805-5

**Published:** 2023-05-10

**Authors:** M. N. Gnanapragasam, A. Planutis, J. A. Glassberg, J. J. Bieker

**Affiliations:** 1 Department of Cell, Developmental, and Regenerative Biology, Mount Sinai School of Medicine, One Gustave L. Levy Place, Box 1020, New York, NY 10029 USA; 2 Department of Emergency Medicine, Hematology and Medical Oncology, Mount Sinai School of Medicine, New York, NY USA; 3 Black Family Stem Cell Institute, Mount Sinai School of Medicine, New York, USA; 4 Tisch Cancer Institute, Mount Sinai School of Medicine, New York, USA; 5 Mindich Child Health and Development Institute, Mount Sinai School of Medicine, New York, NY USA; 6 Present Address: Department of Biological, Geological, and Environmental Sciences, Center for Gene Regulation in Health and Disease, Cleveland State University, Cleveland, OH USA

**Keywords:** Transcriptional regulatory elements, Gene expression, Gene regulation, Genetics research

## Abstract

The onset of erythropoiesis is under strict developmental control, with direct and indirect inputs influencing its derivation from the hematopoietic stem cell. A major regulator of this transition is KLF1/EKLF, a zinc finger transcription factor that plays a global role in all aspects of erythropoiesis. Here, we have identified a short, conserved enhancer element in KLF1 intron 1 that is important for establishing optimal levels of KLF1 in mouse and human cells. Chromatin accessibility of this site exhibits cell-type specificity and is under developmental control during the differentiation of human CD34+ cells towards the erythroid lineage. This site binds GATA1, SMAD1, TAL1, and ETV6. In vivo editing of this region in cell lines and primary cells reduces KLF1 expression quantitatively. However, we find that, similar to observations seen in pedigrees of families with KLF1 mutations, downstream effects are variable, suggesting that the global architecture of the site is buffered towards keeping the KLF1 genetic region in an active state. We propose that modification of intron 1 in both alleles is not equivalent to complete loss of function of one allele.

## Introduction

Hematopoietic stem cells are the source of all the mammalian blood lineages, each arising following a process of gradual restriction of downstream cellular potencies^1^. One of these lineages is erythropoiesis, which gives rise to the red blood cells that make up > 80% of cells in the human body. Critical for this progression are controlling transcription factors, of which Erythroid Krüppel-like Factor (EKLF/KLF1)^2^ plays a global role in all aspects of red blood cell formation^3–6^. KLF1 contains three C2H2 zinc fingers that precisely recognize its cognate DNA site (5′CCMCRCCCN3′) at target genes, enabling a high level of discrimination among similar sites^7^. It performs this function by binding to its cognate DNA element, interacting with the basal transcription machinery, and recruiting chromatin remodeling proteins and histone modifiers^8–10^. KLF1 integration of these signals leads to establishment of the correct 3-dimensional structure necessary for optimal target gene transcription^11^. Regulated expression of KLF1 target genes are coordinated within ~ 40–60 nuclear transcription factories^12^.

Ablation of *KLF1* leads to embryonic lethality in the mouse precisely at the time of the switch to adult β-globin^13,14^. This is only one of many changes that occur in the red cell in its absence, such that KLF1 is now recognized as a global regulator of all aspects of erythropoiesis^3,15,16^. The range of phenotypic/clinical parameters that follow from its genetic modulation in the human has become extensively catalogued^3,4^. *KLF1* haploinsufficiency leads to benign outcomes (e.g., lower expression of CD44 and Lu/BCAM antigen), but compound heterozygosity can lead to more serious problems such as microcytic hypochromic anemia^17^ and pyruvate kinase deficiency/chronic non-spherocytic hemolytic anemia^18–20^ or even to *hydrops fetalis*^21^. Some mutations lead to a dominant phenotype such as seen in congenital dyserythropoietic anemia type IV^22–24^.

Clinical parameters are affected when one *KLF1* allele is inactive, as *KLF1* gene variants are associated with altered red cell indices^25–28^. Of particular note is the loss of β-like globin switching mechanisms, leading to HPFH (originally mapped in a Maltese family^29^) and also to persistence of embryonic globin^18^. There are also benign but significant changes in mean cell volume (MCV) and mean corpuscular hemoglobin (MCH) (both lower), or in zinc protoporphyrin (ZnPP) and reticulocyte count (RTC) (higher). These changes enable identification of *KLF1* mutation carriers^3,4,30^, and it is of considerable interest that allelic distribution of *KLF1* mutations are dramatically higher in regions of endemic β-thalassemia^31^, where the dysregulation of γ-globin expression is of clear clinical benefit. A case report of β^o^/β^o^ thalassemic twins highlights the remarkable clinical difference in anemia and transfusion dependence that follows from loss of one *KLF1* allele^32^. Collectively these data strongly suggest that a fine-tuned understanding of KLF1 promoter regulatory mechanisms could enable directed modulation of KLF1 levels and provide a significant benefit to thalassemic and sickle cell patients.

*KLF1* control elements have been clearly established in vitro^33^ and in vivo^34–37^. These studies have delineated a relatively simple layout of a short (~ 1 kB) region adjacent to the transcriptional start site containing erythroid-specific hypersensitive (EHS1 and 2) upstream enhancer elements that bind hematopoietic general and tissue-restricted regulators^38–41^ and respond to BMP4 signaling. Coupled to this, an enhancer element in intron 1 plays a critical role in establishing its maximal expression level^37^. Epigenetic analyses have determined that the DNA methylation and hydroxymethylation modification status of these controlling regions are important for KLF1 expression control^42–44^.

We recently analyzed the *KLF1* transcription unit for variability across of a range of leukemic and malignant erythroid subsets and found only common SNPs^27^. In the present study, evaluation of the KLF1 transcription unit in Juvenile Myelomonocytic Leukemia (JMML) samples enabled us to more precisely investigate the role of a conserved sequence in intron 1 in modulating expression of *KLF1*.

## Results

### Identification of a mutation at the intronic enhancer region of *KLF1* in a JMML patient

**Figure 1:**
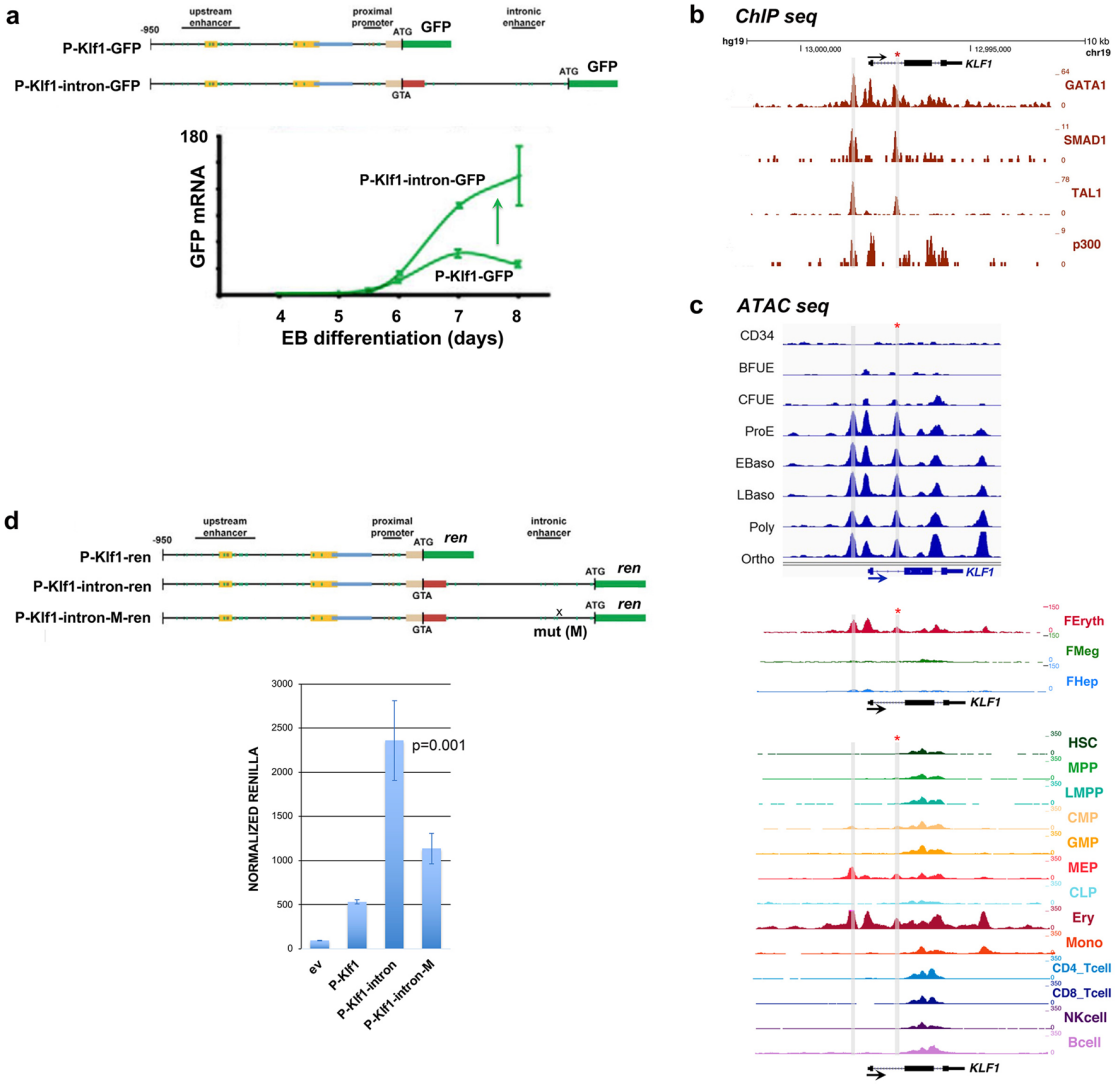
Analysis of the *KLF1* intronic enhancer. (**a**) Reanalysis of data from a study^37^ on the mouse *KLF1* promoter. Constructs containing the *KLF1* promoter or one that additionally includes intron 1 were stably integrated into mouse ES cells adjacent to a GFP reporter, yielding two stable ES lines (“P-Klf1-GFP” and “P-Klf1-intron-GFP”). *Top*: Locations of the mapped upstream enhancer, proximal promoter, and intronic enhancer are as indicated. *Bottom*: ES cells from each line were differentiated to EBs for the indicated number of days, and samples were quantitatively analyzed for expression of exogenous reporter (green) to monitor the effect of intron 1 inclusion. Data is from analysis of biological triplicates. Endogenous KLF1 expression was also monitored in the same samples to show that its onset/expression is similar in both sets (Fig. S5). (**b**) Genome browser data aligned at the human *KLF1* genomic region (https://main.genome-browser.bx.psu.edu/index.html). Shaded bars show the locations of the enhancer upstream of the gene and of the intron 1 site within the gene (also marked with an asterisk). In vivo GATA1, SMAD1, TAL1, and P300 binding identified from ChIP-seq analyses of erythroid cells are shown. (**c**) Genome browser data showing onset of chromatin accessibility as monitored by ATAC-seq analyses of primary human cells: top, during erythroid differentiation from sorted adult human CD34+ cells^49^; middle, from erythroid, megakaryocyte, and hepatic fetal liver cells^52^; bottom, from adult human hematopoietic subpopulations^50^. Shaded bars are aligned with and are as in (**b**). (**d**) Reporter assay of various renilla reporter constructs after transfection into human JK1 cells. Top: Schematic of constructs containing the *KLF1* promoter (as in (**a**)) are shown. The location of the intron 1 point mutant introduced into P-Klf1-intron-Ren is shown (“M”). Bottom: Results of the assay, showing high renilla levels from the promoter alone that are further stimulated by inclusion of the intron (data from two separate DNA preparations, each performed in triplicate); however, these levels are decreased when the point mutant variant is used (data from three separate DNA preparations, each performed in triplicate). Normalization is to a co-transfected luciferase plasmid, and data is an average of triplicate samples from each DNA preparation.

We sought to investigate *KLF1* gene status in cells from patients with JMML, an early childhood myeloproliferative/myelodysplastic disease that is a clonal disorder of pluripotent stem cells^45–48^. They are also thrombocytopenic and exhibit hepatosplenomegaly^45^. We sequenced the complete *KLF1* transcription unit^27^ in genomic DNA from 20 JMML patient samples to search for mutations^48^. We identified three changes within the samples, two of which are in an upstream region just beyond the critical EHS1 enhancer^39,40^ (Table S1, Fig. S1a). Of particular interest, the third sample (HM3554) contains a single heterozygous base mutation within a highly conserved region of intron 1 that is not a SNP (Table S1, Fig. S1b). This change did not appear in our previous analysis of over 5000 genomes from direct sequencing and from public databases^27^. This mutation is located near the intronic enhancer region we had previously discovered in the mouse^37^. Inclusion of this intron leads to a threefold increase in linked reporter activity beyond that seen with the 5′-promoter alone, as assayed in differentiating embryoid bodies derived from mouse embryonic stem cells (Fig. 1a).

A closer analysis reveals that the mutation is located within a sequence that is highly conserved across mammalian species, and directly adjacent to previously identified GATA and SMAD recognition elements in the mouse^37^ (Fig. S1b). This region binds GATA1, SMAD1, and TAL1 transcription factors also in human erythroid cells (Fig. 1b), and the increase in chromatin accessibility during human hematopoiesis, development, and erythroid differentiation as judged by ATAC seq analysis is regulated and centered at that site (along with that of the previously-described upstream promoter/enhancer) (Fig. 1c)^49–52^. These data suggest that the intron 1 site mutated in the JMML patient is within a functionally important and restricted genetic element that is critical for optimal mammalian KLF1 expression.

### Reporter assays demonstrate the relevance of the intronic enhancer mutation on KLF1 expression

We performed reporter assays in transfected JK1 human erythroleukemia cell line to address the importance of the intron 1 mutation. This was assessed by transfection of renilla reporters driven by wild type or site-directed mutated *KLF1* promoter and intronic regions (design based on^37^). Figure 1d shows that, as expected from the murine data, inclusion of intron 1 increases the activity of the promoter ~ 4-fold. However, this increase is reduced by 50% when performing the same assay using a reporter with the intron 1 single nucleotide mutation, verifying its importance. Performing the same assay with the two upstream promoter changes had little effect (Fig. S2).

### Genome editing at the mutated intronic region in the KU812 human erythroleukemia cell line leads to reduced KLF1 expression

**Figure 2:**
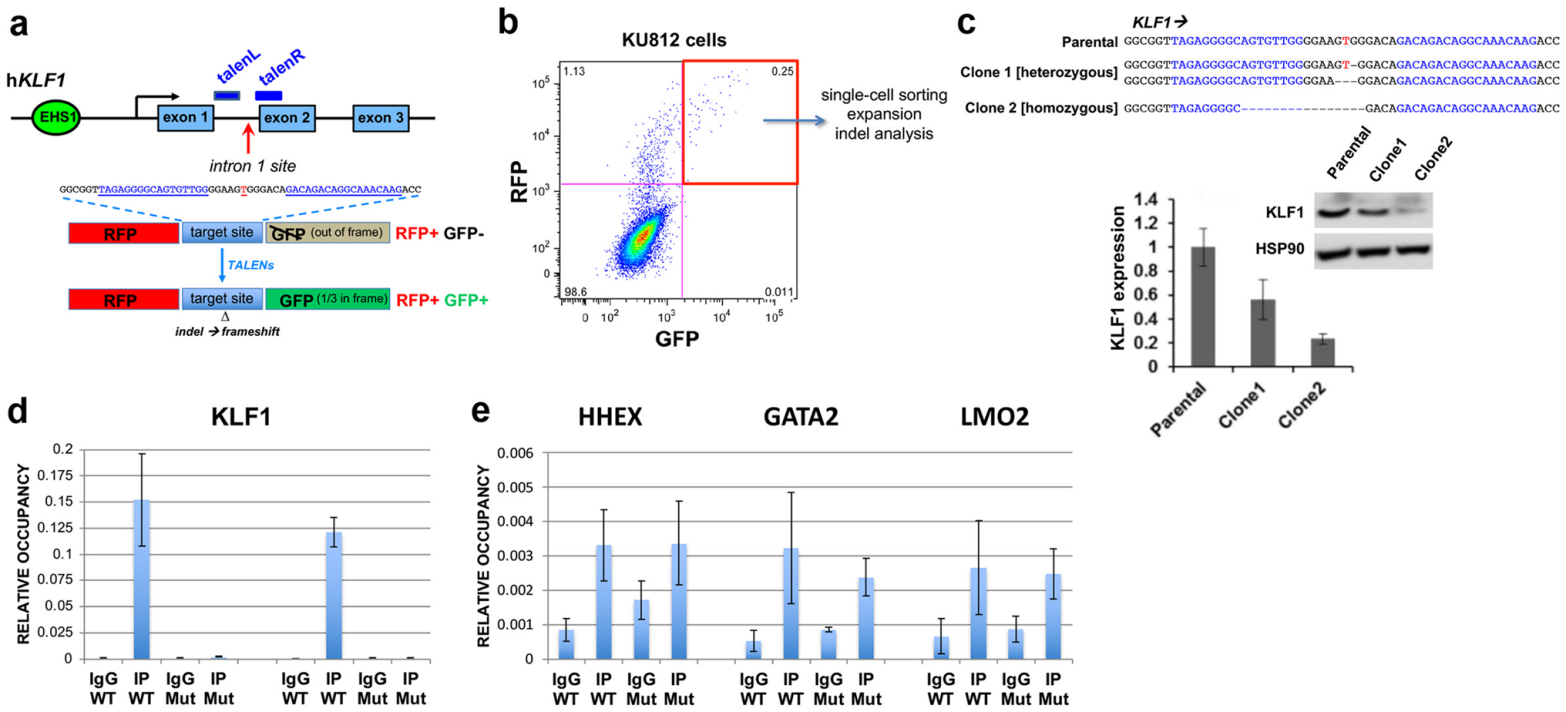
TALEN-mediated indel generation and analysis of KU812 cells. (**a**) *Top*, schematic of the location of the two TALEN arms (blue) in the context of the *KLF1* gene and the intron 1 mutation site (red). Exact sequence is shown. *Bottom*, schematic of parental^55^ and modified constructs containing RFP (in frame) and GFP (out of frame) reporters separated by a target site into which the intron 1 sequence (with the left and right TALEN sites) is incorporated. Successful transfection and activity of the dual TALENs is predicted to yield an indel leading to a frameshift and resultant in-frame expression of GFP (1 out of 3 chance). (**b**) Data is shown from the transfection of KU812 cells. Selection of RFP+/GFP+ positive cells after cotransfection with left and right TALEN arms greatly increases the chance of finding a clone with the desired indel, even at low frequency. Clones are generated by single-cell sorting and expansion. (**c**) Genomic sequence analysis of parental and two KU812 subclones (“clone 1” and “clone 2”) containing directed indels at *KLF1* intron 1; blue are the TALEN sequences, red is the target JMML site as a reference point. Homo- or heterozygous deletions are as indicated. Analyses of the two indel KU812 cell clones compared to parental cells are shown after a check of KLF1 RNA expression and western blot assessment (uncropped blot is in Fig. S3) of KLF1 and HSP90 protein (insert). (**d**) ChIP analysis of specific TEL/ETV6 binding in WT or mutant (clone 2, “Mut”) KU812 cells. ETV6 occupancy at the *KLF1* intron 1 site, monitored by two different primer pairs, show a positive signal only in WT cells. (**e**) Positive control targets known to bind ETV6 show signals in all cases (HHEX, GATA2, LMO2 based on^114^) in WT or mutant cells. For (**d**) and (**e**), multiple samples were analyzed, each in triplicate.

We next addressed whether the sequence surrounding the mutation is critical for hKLF1 transcription by using TALEN directed-nuclease technology^53,54^ to delete the site within the *KLF1* genome in human cells (Fig. 2a, Table S2), and monitored KLF1 levels. We were aided in the selection process by the use of a cotransfected RFP/GFP reporter^55^ (Fig. 2a). Using this system we transfected human KU812 cells which, although of low transfection efficiency via lipofection, enabled us to select for double positive RFP+/GFP+ cells (Fig. 2b).

The clones encompassed homo- and heterozygous deletions of 3–15 nt centered at the intron 1 mutation site (e.g., Fig. 2c), indicating successful attainment of genomic editing. Clones of non talen-treated parental cells were also obtained to serve as a control to avoid any variables from clonal selection. Western and qRT-PCR analysis was performed for clone 1 (heterozygous deletion), clone 2 (homozygous deletion), and one of the parental clones (Fig. 2c). Western analysis shows that KLF1 protein levels are quantitatively reduced in clone 1 and more reduced in clone 2 compared to a parental clone, effects that mirror the KLF1 transcript expression (Fig. 2c).

These results enable us to sublocalize the functionally critical sequence from within the original large 0.9 kB intron 1 to a small ~ 15 bp region surrounding the intron 1 site mutation, and suggest that a circumscribed deletion centered on this mutation site within normal cells will quantitatively decrease, but not ablate, KLF1 expression.

### Identification of TEL/ETV6 protein interaction with the *KLF1* intron 1 region

Given the high level of sequence conservation at this site (Fig. S1b), transcription factor interactions may be predicted to be negatively affected by the mutation. We used the UniPROBE factor interaction prediction analysis to identify binding sites that are directly disrupted by the intronic mutation^56,57^ coupled to analysis via the NIH Roadmap Epigenomics Consortium (Fig. S4). We find that the intron region surrounding the mutated site is enriched for binding sites for the ETS family of proteins^58,59^ that interact with a consensus motif known to be important for the transition from stem to erythroid cells^60^. The single base intron 1 mutation is predicted to disrupt the binding for these proteins (the “T” in the sequence GGAAGT, mutated to a “C” in the present case, is particularly critical in all cases)^57^. Exclusion of those transcription factors not expressed in erythroid cells (analysis via ErythronDB^61^ and BioGPS^62^) leaves only a small subset as possible contenders for positive binding such as ERG, ETV6, and ELF2. Of particular interest is TEL/ETV6, a protein that, similar to KLF1, has known roles in enhancing erythroid differentiation. An in vitro binding site selection strategy for ETV6 verifies the importance of a “T” at the position of interest^58^. In vivo ChIP analyses of WT and intron 1-edited KU812 cells show that TEL/ETV6 binds to this site, and that its binding is abrogated when the target site is mutated (Fig. 2d). TEL/ETV6 binding to three control target sites are not affected in cells containing the mutation at the KLF1 site (Fig. 2e). We conclude that the TEL/ETV6 protein joins GATA1/SMAD/TAL1 in forming an optimal protein/DNA complex at this critical site within intron 1 of the *KLF1* gene.

### Genome editing of KLF1 intron 1 in human CD34+ cells

**Figure 3:**
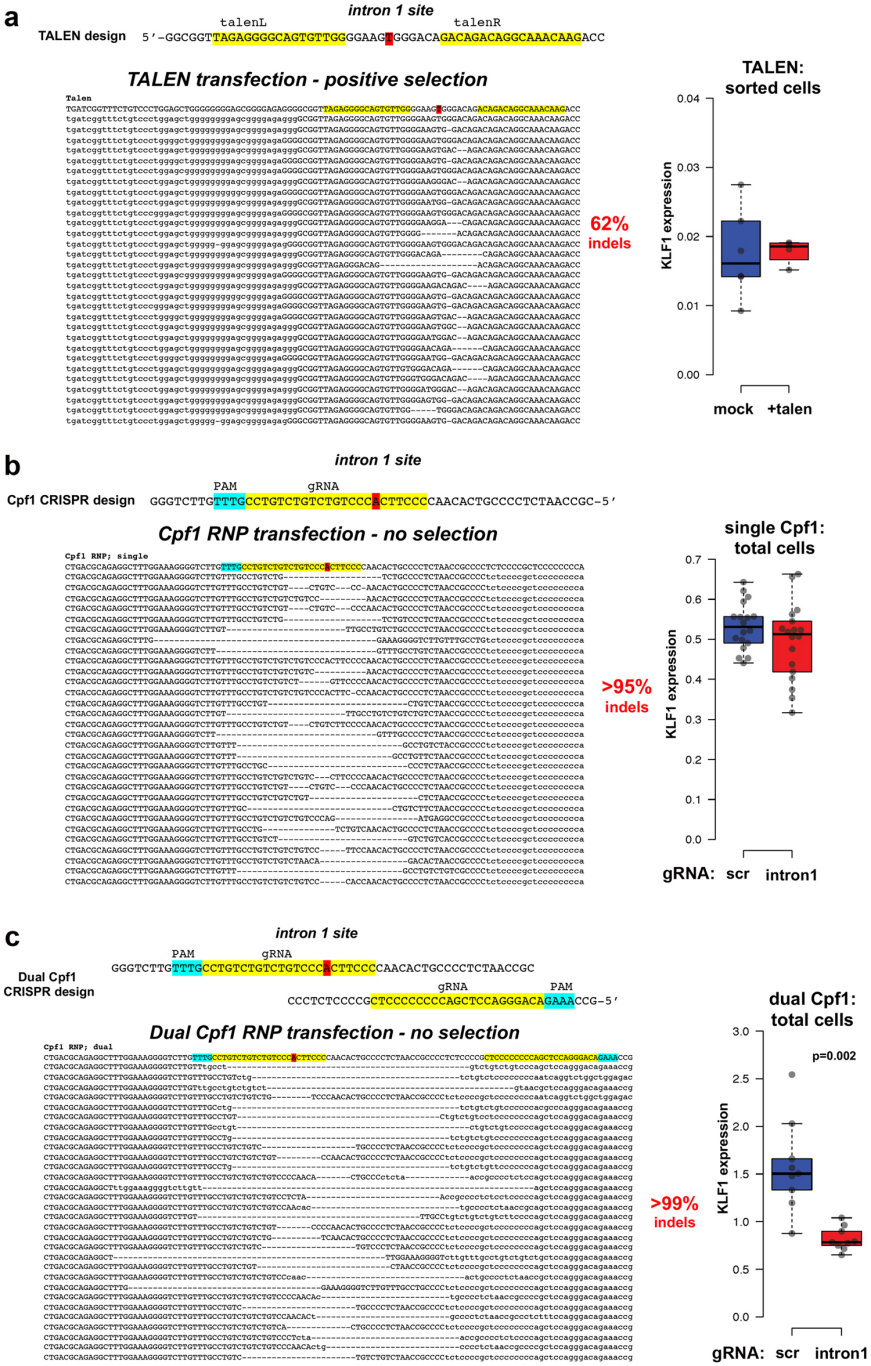
Gene editing and analysis of primary human cells. Human CD34+ cells were transfected, differentiated towards the erythroid lineage with a three-phase protocol, and analyzed by deep sequencing of the *KLF1* intron 1 region. Indels were identified, and the top 30 are shown in each case along with the total indel percentage in the population (one representative example from each test is shown). The location of the JMML point mutation (marked in red at the top) serves as a reference point for each design. KLF1 mRNA expression was monitored by RT-qPCR and shown on the right. (**a**) Cells were transfected with the left and right TALEN arms (indicated at the top in yellow) along with the co-transfected reporter. The RFP+/GFP+ pool was sorted, expanded, and differentiated prior to NGS genomic DNA analysis. KLF1 expression levels are not significantly different (n = 4–6 each). (**b**) Cells were transfected with an RNP consisting of Cpf1 protein and gRNA (indicated at the top in yellow, with the PAM sequence in blue). The negative control was cells transfected with an RNP containing scrambled gRNA. The cells were expanded and differentiated prior to NGS genomic DNA analysis. NGS of the scrambled control cells showed no changes (not shown). Although there is a range of KLF1 expression after editing, levels are not significantly different from the scrambled control (n = 18 each). (**c**) Cells were transfected with a 50/50 mix of RNPs consisting of Cpf1 protein and two gRNAs (indicated at the top in yellow, with their PAM sequences in blue). The negative control was cells transfected with an RNP containing scrambled gRNA. The cells were expanded and differentiated prior to NGS genomic DNA analysis. NGS of the scrambled control cells showed no changes (not shown). KLF1 expression is down twofold in the samples dual-edited samples (n = 9 each).

To test whether quantitative knock down of KLF1 expression could be attained by editing of intron 1 in primary cells, we expanded human CD34+ cells and transfected them with the verified TALEN arms (along with the RFP/GFP reporter), followed by differentiation towards the erythroid lineage using a three-step protocol^63–66^. Positively-transfected cells were selected (as before) and cultured as a pool of cells. Sorted cells from transfections without the TALEN arms served as our unaltered control. Deep sequencing of the positive pool indicates that over 60% of the cells are edited at the expected deletion site that overlaps the intron 1 mutation (Fig. 3a). Although encouraging, we found the resultant small deletions had little effect on KLF1 expression, possibly due to the fact that our efficiency was not near 100%, viability was not good after the DNA transfection, and at most we should have detected a 30% drop in expression (50% effect x ~ 60% cells with deletion).

During the initial design of these studies, we had not been able to use a CRISPR/Cas approach due to the inherent requirements of Cas9 gRNA, whose PAM sequence (NGG) limited the target sequences to regions not near the site of interest in intron 1. However, in the interim Cpf1 (aka Cas12a) was discovered and developed as a viable alternate^67,68^. Its merits in the present situation are that its PAM sequence (TTTV) is significantly different from that of Cas9, and that the *Acidaminococcus *sp. variant has been engineered for increased activity and fidelity^69^. This novel recognition sequence enabled us to design a Cpf1 gRNA that directly overlaps the site of interest and exhibits no predicted off-targets even with up to 2 mismatches (Table S3) (CRISPOR program^70^). We also opted to use an RNP-based approach to enable entry of the Cpf1/gRNA complex into CD34+ cells at high efficiency while retaining high cell viability^66,71–73^. We tested this new design/approach, and indeed find that, even in the absence of selection, > 95% of the cells are edited (Fig. 3b). Use of a scrambled Cpf1/gRNA control routinely gives close to zero editing. The deletions at the region of interest are larger than had been observed with TALENs, but surprisingly we still do not see a significant effect on KLF1 levels on average even though the range of effect was wide (i.e., a subset of the data points were decreased compared to the control).

We next considered whether the Cpf1-directed deletion at the *KLF1* intron 1 site might be too limited. Although one might predict that disruption of transcription factor binding should have a major effect on activity of enhancer function, it has been observed that removal of a single site in vivo can have a surprisingly minor effect (concepts discussed in^74^), as any associated ‘enhanceosome’ components may still form if those protein–protein interactions are strong, and if there are other adjacent DNA binding sites that retain recruitment and cooperativity of other components in the full complex. Indeed, the *KLF1* gene forms an extended 3D complex with multiple enhancers in its vicinity^75^. We inspected the sequence surrounding the original Cpf1 position and found an additional Cpf1 PAM-sequence target site ~ 35 bp away that also exhibits no predicted off-targets even with up to 2 mismatches (Table S3) (CRISPOR program^70^). We postulated that introduction of both Cpf1 gRNA sequences might enable a larger deletion region to be generated. This dual Cpf1 approach was tested. Again we attain high efficiency of editing in the absence of selection (> 99%), but we now create a larger deletion and produce the desired 50% drop in KLF1 levels (Fig. 3c). This suggests that focused disruption of the ETV6 site is not sufficient, but rather that deletion of a slightly larger region, likely containing other important binding sites [e.g., MYB, SMAD, and GATA proteins (Figs. S1B ad S4)], is needed to more reliably disrupt the enhancing activity of intron 1. Importantly, this verifies the importance of the intron 1 region for optimal KLF1 expression in vivo in primary cells and set the stage for analysis of its downstream effects.

### Gene expression analyses of *KLF1*-edited CD34+ cell-derived erythroid cells

**Figure 4:**
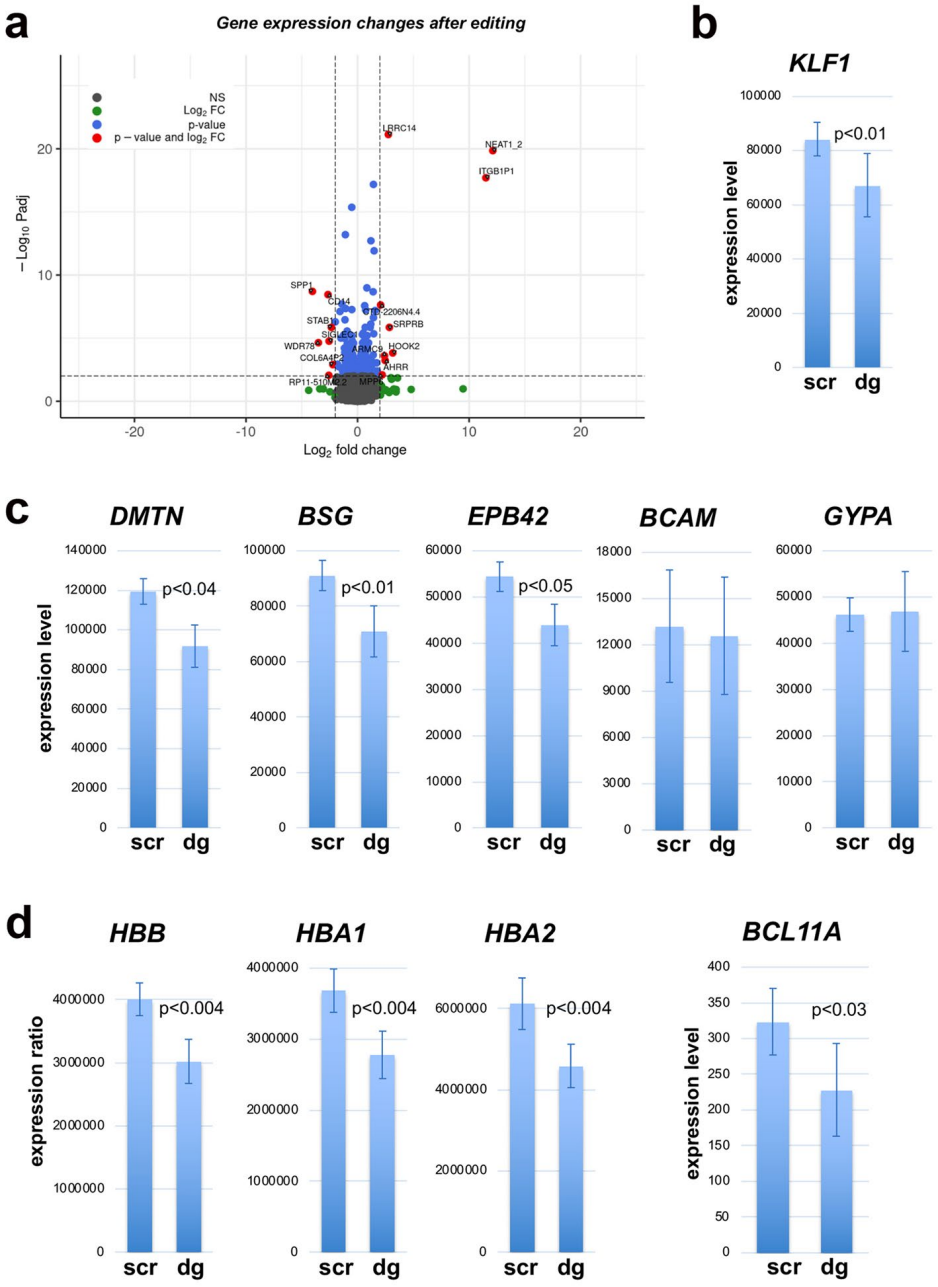
RNA seq analysis of edited CD34+ cell-derived erythroid cells. (**a**) Volcano plot of RNA expression data from control compared to intron 1-edited CD34+ cells is shown based on analysis of biological triplicate samples harvested at d18 of the three-phase differentiation protocol. Black dots represent genes not changed in expression, green are changed but not significantly, blue are significantly changed but less than |log2| = 1.0, and red are significantly changed and |log2| ≥ 1.0. Some genes in this last category have been identified in the figure. These genes are listed in Tables S5 and S6. (**b**–**d**) RNA seq expression data from control (transfected with Cpf1 RNP containing scrambled gRNA; ‘scr’) compared to that from intron 1-edited (transfected with a Cpf1 RNP containing a dual guide mix of intron 1-directed gRNA as in Fig. 3c; ‘dg’). Statistical analysis is from the DESeq2 files (as in (**a**)). The source file for these genes is Table S7. (**b**) Expression of KLF1. (**c**) Expression of genes anticipated to be affected by drop in KLF1 levels. (**d**) Expression of genes related to β- and α-globin gene regulation, including the BCL11A repressor.

To understand the genetic effects of *KLF1* intron 1 gene editing, we undertook two sets of analyses. First, we compared expansion/differentiation properties of control vs intron 1-edited cells and find no significant changes during expansion, and perhaps a minor but still not significant effect on the extent of differentiation (Fig. S6), properties consistent with that from haploinsufficient individuals^76^. Second, we performed RNA seq on triplicate samples derived from the larger dual guide-edited or control CD34+ cells isolated at the end of the differentiation protocol. A comparative analysis of these two sets of data shows that the editing has a circumscribed/limited effect, with a relatively small number of genes (~ 50 either up/down, p < 0.05, |log2| ≥ 1) altered in their expression (Fig. 4a). Some of the downregulated genes bind KLF1 in vivo via its cognate binding sequence and occasionally overlap with GATA1 binding^77,78^. The limited effect may be due to the unexpected low outcome of the deletion on KLF1 expression (i.e., 20% rather than 50%) (Fig. 4b). The variation in expression of KLF1 after deletion within intron 1 using the same dual guide approach but with different CD34+ cell sources (comparison of Figs. 3c and 4c, but also foreshadowed by the large range of effect in Fig. 3b) led us to examine whether upstream regulators of *KLF1* might variably be increased to compensate^10,39^. However, there is no coherent increase in expression levels of proteins known to coordinately interact with the critical *KLF1* upstream enhancer element (EHS1) within the edited cells (Fig. S7). Given that adults that have one mutated allele actually express 50% of the normal KLF1 level^29,76^, our results imply that haploinsufficiency by virtue of deletion of intron 1 across both alleles is not equivalent to haploinsufficiency from total functional loss of one allele. This suggests the *KLF1* locus is buffered against large changes in expression (more fully discussed below).

In spite of this, a number of cellular/structural genes anticipated to be altered in expression based on analyses of *KLF1* haploinsufficient individuals^79^ are affected and include *DMTN*, *BSG*, and *EPB42* (Fig. 4c). However, other targets such as *BCAM* and *GYPA* are not affected (Fig. 4c).

With respect to globin regulation, unsurprisingly, expression of the adult β- and α-globin genes are decreased in the edited cells (Fig. 4d). Even though the upstream γ-globin repressor *BCL11A* (that is directly regulated by KLF1^29,36^) is also decreased in expression (Fig. 4d), levels of other repressors are not significantly affected, leading to minimal effects on γ- and ε-globin expression (Fig. S8). As a control, we edited the + 58 region of the *BCL11A* gene^66^ with our methodology and find significant increases in γ-globin expression and the γ/(γ + β) ratio (Fig. S9).

Collectively, these studies show that the dual guide design directed at the intron 1 enhancer element leads to quantitative disruption of KLF1 expression in differentiating human CD34+ cells, and that even less than a two-fold drop leads to measurable changes in expression of its target genes. However, unlike the initial results in cell lines, reproducible knock-down of expression in primary cells requires additional genetic manipulation.

## Discussion

Genetic studies in mice and humans have been critical for discovering that two-fold changes in KLF1 expression can lead to clinically useful changes in gene expression. KLF1 as a therapeutic target has largely been abandoned because its absence creates lethal problems for the red cell^6,13,14,21^. However, the present design stresses the importance of precise quantitative expression control and is based on the observation that a haploinsufficient level of KLF1 is benign, and that manipulations that mimic this effect could have therapeutic benefit because only select targets are dramatically affected by the 50% drop in expression^8,79–81^. Cultured erythroblasts from haploinsufficient individuals proceed through renewal and differentiation stages equivalent to wild type, and overall chromatin accessibility is unchanged^76^. An alternate gene modification would be to disrupt the coding sequence, but a recent study that depleted KLF1 in this way in human CD34+ cells led to many genes dysregulated in their expression^82^.

The overall effect on KLF1 expression of deleting the intron 1 site via the dual Cpf1/gRNA approach remained relatively minor when tested in primary cells; this was unexpected, given that the evidence from the reporter transfections and from the Talen cell line editing had shown that even a directed mutation/small deletion exerts an effect on reporter or endogenous KLF1 transcription. However, transfected DNA begins in a non-chromatinized conformation when introduced into the cell, and leukemic cell lines are generally dysregulated; hence the primary cell approach is the most stringent and informative way to mimic the in vivo cellular and epigenetic environments. The quantitative effect of the indel on KLF1 transcription in primary cells, even at a level of 20%, was precisely tracked by a remarkably similar quantitative reduction in expression of a subset of its erythroid targets. This illuminates the extreme level of precision in transcriptional control that, based on KLF1 regulatory properties^83^, could include precisely attenuated transcriptional pause/release mechanisms for which these target genes may be extremely sensitive.

The inclusion of ETV6 into a positive regulatory role is perhaps unusual, as it has been typecast as a transcriptional repressor. However its molecular role is complex, and in addition a recent study shows that KLF1 levels are decreased in homozygous mutant *ETV6* iPS cells that were established as a model of inherited thrombocytopenia^84^. These cells also exhibited decreased erythroid expansion capability and erythroid colony counts.

With respect to future applications of our approach, KLF1 expression is highly restricted to erythropoietic organs, as evidenced during development^85,86^, in the adult^2^, and during hematopoiesis^87–89^. The highly restricted nature of its expression is a crucial property for its potential use as a gene therapy target, as quantitative alteration of its levels is predicted to limit its effect to erythroid-related cells of interest, a unique situation that avoids the complexities that arise in other potential targets that are more generally expressed and required^90–94^.

A perplexing observation in the field is the extensive variation in HbF levels seen in individuals containing the same monoallelic *KLF1* mutation, even within the same family pedigree^16,29,95^. This was also seen in our own studies (comparison of the dual gRNA data between Figs. 3 and 4). This is thought to be due to variable penetrance and microvariation of KLF1 expression as contributed by additional mutations on the remaining WT allele^76,95^. Our data suggest that the overall architecture of the *KLF1* genomic region is buffered against localized changes having a substantive effect on its expression. This idea is in tune with recent observations demonstrating exactly this phenomenon and explained by the establishment of nested, two-layer networks of enhancers, some of which are distantly located and in non-coding regions, that achieve the net effect of protection against chromosomal perturbations^96,97^. Consistent with this idea, the *KLF1* transcription unit is known to be located in the midst of multiple interacting domains^75^, and the *KLF1* promoter is one of a small subset of tissue-restricted genes whose expression is dependent on multiple regulatory elements^98^. As a result, reproducible and precise management of KLF1 levels may require additional manipulation(s) in coordination with directed changes at intron 1.

## Methods

KU812 and JK1 cells lines have been described^99^. We initially considered mutating the intron 1 region in HUDEP2 cells^100^, as they have been widely utilized for human erythroid studies. However, we obtained aberrant results with all our directed mutagenesis attempts at the *KLF1* genomic region. Karyotype analysis revealed genomic instability along with trisomy 19 (not shown), which is the genomic location for *KLF1*. As a result, we switched to using JK1 and/or KU812 human leukemia cell lines for our initial set of gene editing analyses, as both of these appear more stable and, unlike K562, also express KLF1^101,102^ (discussed in^27^) and thus more likely to exhibit positive control of an exogenous KLF1 promoter/enhancer construct. Patient DNA samples are not publicly available but were as previously published^48^ and obtained from Dr M Loh (UCSF), whose studies were approved by the UCSF Committee on Human Research. All participants or their guardians provided informed consent in accordance with the Declaration of Helsinki. As a result, samples and data were analyzed anonymously. Genomic sequencing using primers spanning the complete KLF1 transcription unit, including the upstream promoter, was performed in both directions as previously described^27^.

Reporter assays were performed after cotransfection of renilla reporter constructs into JK1 cells using X-tremegene HP transfection reagent (Sigma) along with a luciferase expressing plasmid for normalization^103^. Assays were performed using the dual luciferase kit (Promega) according to manufacturer’s instructions. Mutagenesis was performed with the Quick-change kit (Stratagene).

Mouse embryonic stem cell culture and differentiation into embryoid bodies was performed as described^37^ using cell lines generated by targeting the P-Klf1-GFP or P-Klf1-intron-GFP constructs into the site-specific Ainv18 homing site^37^. These contain a single copy, unidirectionally inserted sequence into the same site and avoids random integration.

Browser data showing ATAC-seq analyses of primary human cells were derived from^49,50,52^. Data for ChIP transcription factor binding was selected from the Penn State University VISION hematopoiesis human GRCh37/hg19 genome browser (https://main.genome-browser.bx.psu.edu/index.html), focusing on results using CD34+ or bone marrow derived erythroid cells.

Human CD34+ cells were purchased from AllCells or obtained from the Yale Cooperative Center of Excellence in Hematology. These were differentiated ex vivo under three-phase protocols, initially based on^63,104^ but then later based on^64–66^. Briefly, CD34+ cells were thawed on day 0 into X-VIVO 15 (Lonza, 04–418Q) supplemented with 100 ng/ml human stem cell factor (SCF), 100 ng/ml human thrombopoietin, and 100 ng/ml recombinant human Flt3-ligand. Transfection was performed before the expansion phase I, and cells were allowed to recover for 24–48 h in X-VIVO media prior to switching to phase I. The base erythroid differentiation medium (EDM) consists of IMDM supplemented with 330 μg/ml holo-human transferrin, 10 μg/ml recombinant human insulin, 2 IU/ml heparin, 5% human Octoplas AB plasma (OctaPharma), 3 IU/ml erythropoietin, 1% l-glutamine, and 1% penicillin/streptomycin. During phase I (days 0–7), EDM was further supplemented with 10E–6 M hydrocortisone, 100 ng/ml human SCF, and 5 ng/ml human IL-3. During phase II (days 7–11), EDM was supplemented with 100 ng/ml human SCF alone. During phase III (days 11–18), EDM had no additional supplements. All analyses were performed after harvesting the cells on day 11 or 18. FACS was performed on an Attune NxT Flow Cytometer using PE-conjugated mouse anti-human CD71 (Cat. 555537) and APC-conjugated mouse anti-human CD235a (Cat. 551336) antibodies from BD Biosciences. FCS Express version 7 software was used for subsequent analysis. Brightfield photography was performed with a Nikon Micro-phot-FX microscope equipped with a Q-Imaging camera.

Cloning of left and right TALEN arms was performed by insertion into a CMV promoter-containing plasmid, downstream of T7 polymerase promoter, HA tag, and NLS sequences, but upstream (in frame) of the Fok1 nuclease and bGH poly(A) signals (PNA Bio Inc). In addition, an RFP/GFP surrogate reporter was synthesized that contained the intron 1 sequence between them as a linker, but out of frame for GFP. Proper nuclease activity after transfection/expression leads to frameshift mutations and expression of GFP, enabling enrichment of a low number of positively transfected cells by sorting for RFP+/GFP+ double positivity (Fig. 2a,b)^55^.

Transfection of TALEN DNAs into KU812 cells was performed using the Neon Transfection System (1450v, 10 ms, 3 pulses), sorted for RFP+/GFP+, and the positive pools were diluted to single cells into 96-well plates. After expansion, DNA was isolated and analyzed by PCR at the region of interest (using sequencing primer pairs^27^) to determine whether any of these clones contained a deletion. Individual clones that were positive were expanded and used for subsequent analyses. Genomic PCR of cell line material used 25 ng purified DNA (Qiagen), followed by direct sequence analysis of the product (Macrogen).

CD34+ cells were transfected with the Amaxa Nucleofector II using program U-008 for TALEN DNA. For RNP we used the Amaxa 4D Nucleofector with program EO-100. Biological replicates (typically three) were each ultimately analyzed in triplicate.

AsCpf1 protein (enhanced^69^) was purchased from IDT. RNPs were freshly formed by mixing 105 pmol of Cpf1 protein with 120 pmol of gRNA (IDT) for 15 at room temperature, and kept on ice until use.

Positive pools of CD34+ cells were analyzed by next generation sequencing (MGH DNA Core Facility) following genomic DNA isolation and PCR. Alternatively, TIDE^105^ or ICE^106^ analysis was performed on these or on KU812 cell DNA samples using sequencing primer pairs^27^.

Predicted off-target effects of the TALEN pair was tested using the PROGNOS analysis program^107^. We found no predicted off-targets when 0, or even 3, mismatches are allowed per arm (Table S2)^107^. Predicted off-targets for the two gRNA designs used the CRISPOR program^70^, which showed no predicted off-targets even allowing for up to 2 mismatches (Table S3).

Sequences of all primers and oligos used for analyses are listed in Table S4. Indels directed at the ‘+58’ region of the *BCL11A* gene were generated by using gRNA #1617 and analyzed using its associated genomic primer pairs^108^.

RNA isolation and RT-qPCR analysis was as previously described^37,109^, and normalized to catalase^108^. RNA seq analysis was performed as described^23^ using NEBNext polyA selection (Cat. # E7490S) and RNA Library Prep Kit (Cat. # E7770L). Sequencing was performed on the Illumina NovaSeq 6000 System at the Icahn School of Medicine Genomics Core Facility. Raw data have been submitted to the Gene Expression Omnibus (GSE223212). The derived data used for the analysis of Fig. 4a (filtered by p < 0.05, |log2| ≥ 1) are listed in Tables S5 (downregulated) and S6 (upregulated); the derived data for the analyses of Fig. 4c–e, Figs. S7, S8 is listed in Table S7 (filtered by removal of padj = NA^110^).

Bioinformatic analysis was performed as described^86^. Statistical analysis and representations were performed in R with package DESeq2^110^. Figures were made with packages ggplot2 and EnhancedVolcano along with other packages^111,112^ (see Table S8 for additional links).

Chromatin immunoprecipitation (based on^113^) of TEL/ETV6 in KU812 cells after cross-linking with 1.0% formaldehyde was performed with a mix of antibodies (containing anti-TEL from Santa Cruz sc-1668335 and sc-8547 along with home-made rabbit polyclonal; generous gifts from Graves and Clark^59^). Chromatin was sonicated using a Sonics Vibra Cell 500 for ten 40-s pulses at 21% amplitude with 1-min intervals. Positive control targets (*HHEX*, *GATA2*, *LMO2*) were chosen based on^114^.

## Supplementary Information

Supplementary Figures.

Supplementary Tables.

Supplementary Table S5.

Supplementary Table S6.

Supplementary Table S7.

## Data Availability

All data generated from RNA Seq is deposited in the Gene Expression Omnibus (GEO) and is publicly available as of the date of publication, accession number GSE223212. The derived data used for the present analyses are listed in Tables S5 (downregulated), S6 (upregulated), and S7.
