# Supplementary material for: Identification of a genomic DNA sequence that quantitatively modulates KLF1 transcription factor expression in differentiating human hematopoietic cells
Source: Sci Rep. 2023 May 10;13:7589. doi: 10.1038/s41598-023-34805-5 (PMC10172341; doi:10.1038/s41598-023-34805-5)
Supplement: Supplementary file 1 — Supplementary Figures. [file 41598_2023_34805_MOESM1_ESM.pdf]

Supplemental Figure S1

a

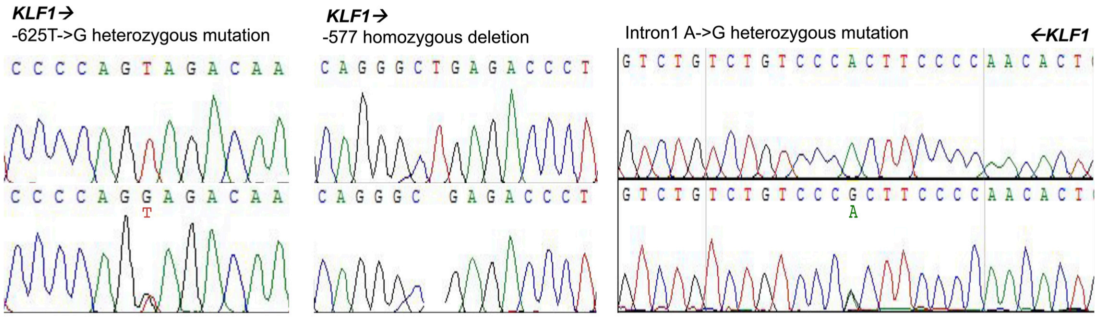

b

Intron1 region

```
Alignment block, 12886210 - 12886404, hg38

Human   gctggacactctgacg-------------cagaggctttggaaa--ggggtcttgtttgcctgtctgtct
Chimp   gctggacactctgacg-------------cagaggctttggaaa--gtggtcttgtttgcctgtctgtct
Rhesus  gctgggtactcttacggggagaggctggccagaggctttggaaa--agggtcctgtttgcctgtctctct
Mouse   gctggaaacaccgttg----------------------------------------gtgcctgt------
Rat     gctggaaacatcgttgggg----------cagg----ttggttg--agggt-ttgcgtgcctat------
Dog     gctggatgccctgatgggggcaggctggccagaggctctggaaa--ggggtcctgcttgcttgtctgtc-
Opossum tcctccagtctagaaagaggggtcaggcctggggtcctgagagataggactttgcttctttgtctctc-

           Ets site                                          Ebf site
Human   gtcccacttccccaacactg-----cccctctaaccgcccctctccccgctccccccccagctccaggga
Chimp   gtcccacttccccaacactg-----cccctctaaccgcccctctccccgct-cccccccagctccaggga
Rhesus  gtcccacttccccaacacta--gccgccctctagccgcccctctcccccct-ctcccccagctccaggga
Mouse   ----catttaccc-acacgg-----ttcccctaaccc---------------ctttctcagctccaga--
Rat     ----catttaccc-acactg-----ttcccctaaccc---------------ccttctcagctcccgg--
Dog     ----cccttgccttacaatgcacactcccccag-------------------caccccaagtccagggc
Opossum ----tattccccaacccctg-----ttgcac---------------------cccatccaccgacatgtg

                         Smad         Smad    Gata/Smad      Gata
Human   cagaaaccgat---caggtctgg---ctggagacaggagataagacttccactatctggaaccacactcg
Chimp   cagaaaccgat---caggtctgg---ctggagacaggagataagacttccactatctggaaccacactcg
Rhesus  cagaagccgat---caggtctgg---ctggagacaggagataagacttccactatctggaaccacacttg
Mouse   c--aactccat---taggtcttg---ctagagac---agctaagacacttacca---agaatagtattc-
Rat     c--aactccat---gaggtcttg---ctagagac---agataagacacttacca---agaacagcattc-
Dog     cagaggccgat----gggtctgg---ctggagacccaagataagactaacattatctggagccacactca
Opossum gaggaaagcattcctgggccctgagactggaaggaacagataagact---gttatctagactcacatatt

           ←KLF1
Human   gggatgggga
Chimp   gggatgggga
Rhesus  gggatgggga
Mouse   aggatgggaga
Rat     aggatgggaga
Dog     ggcatgggga
Opossum gagatggcaag
```

Figure S1. Sequence analysis of mutations identified from the JMML samples.

(a) Sequence traces of regions surrounding -625, -577, and intron 1 mutation locations, showing WT sequence on *top* and mutant sequence *below*, with substitution/deletion as indicated. Orientation of *KLF1* gene is indicated by direction of arrows. The upstream sequences lie outside of the human EHS1 enhancer element that maps from -529 to -378 [39,40].

(b) Conservation of intron 1 region across selected vertebrates, with *KLF1* gene orientation from right to left as indicated. The mutation is indicated in red, as is the putative ETS binding site in yellow. Green are other previously identified conserved regions [37].

Supplemental Figure S2

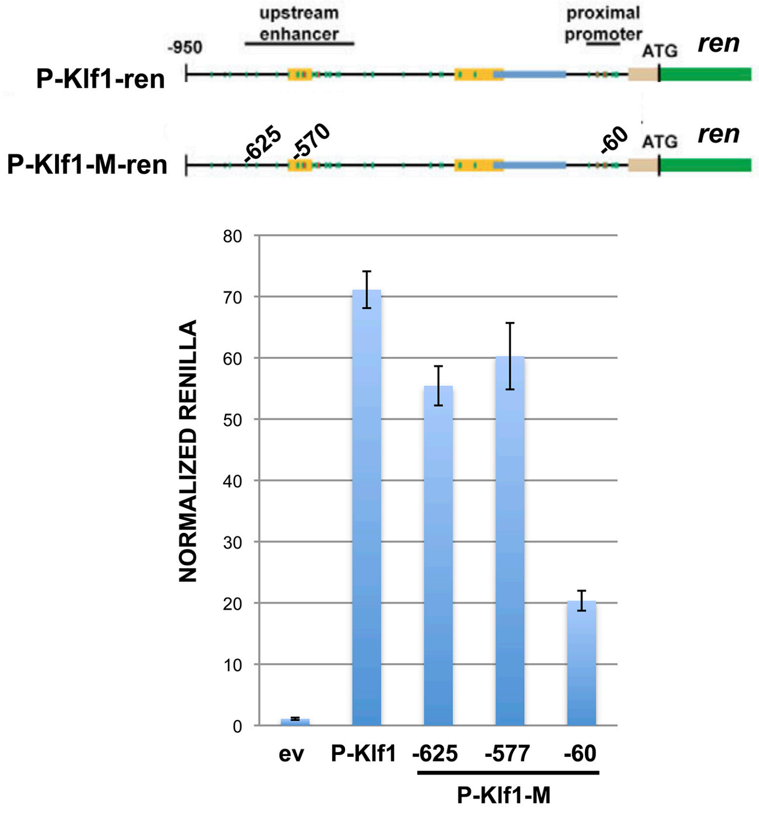

Figure S2. Reporter assay of various renilla reporter constructs after transfection into human JK1 cells.

Top: Schematic of constructs showing the location of the JMML mutants introduced into P-Klf1-intron-Ren ("M").

Bottom: Assay results show that high renilla levels from the promoter are not significantly altered after inclusion of the -625 or -577 mutation; a reporter containing the -60 mutation (located at an important GATA site [38]) is included as a positive control. Normalization is to a co-transfected luciferase plasmid, and data is an average of triplicate samples.

Supplemental Figure S3

Single clone genomic analysis
[lanes from left to right]

Experimental:
1=het deletion
2=off target het
3=no genomic pcr product
4=homo deletion *'clone 2'*
5=het deletion *'clone 1'*

RFP=mock *[parental]*
1 'parental'
2

Control=untransfected
1
2

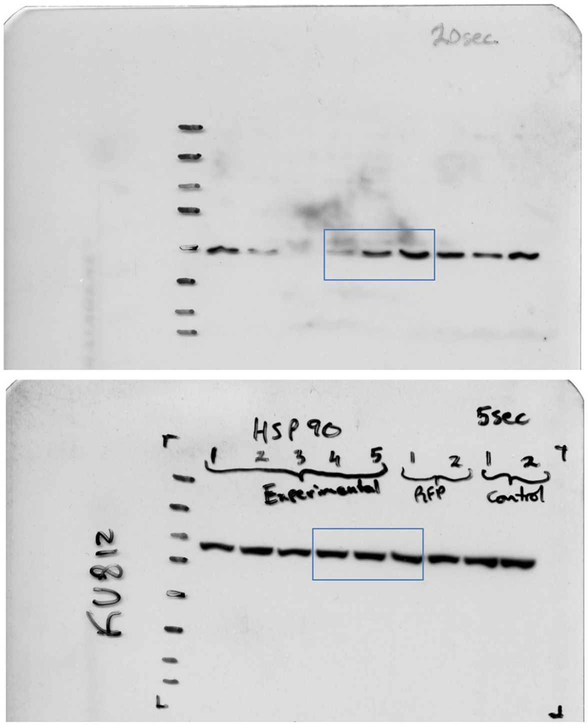

Figure S3. Protein analysis of TALEN-modified clones.
Uncropped blots (*top*, anti KLF1; *bottom*, anti HSP90 control) that provide the data (boxed regions) for the three lanes (parental, clone 1, clone 2) shown in Fig 2c.

Supplemental Figure S4

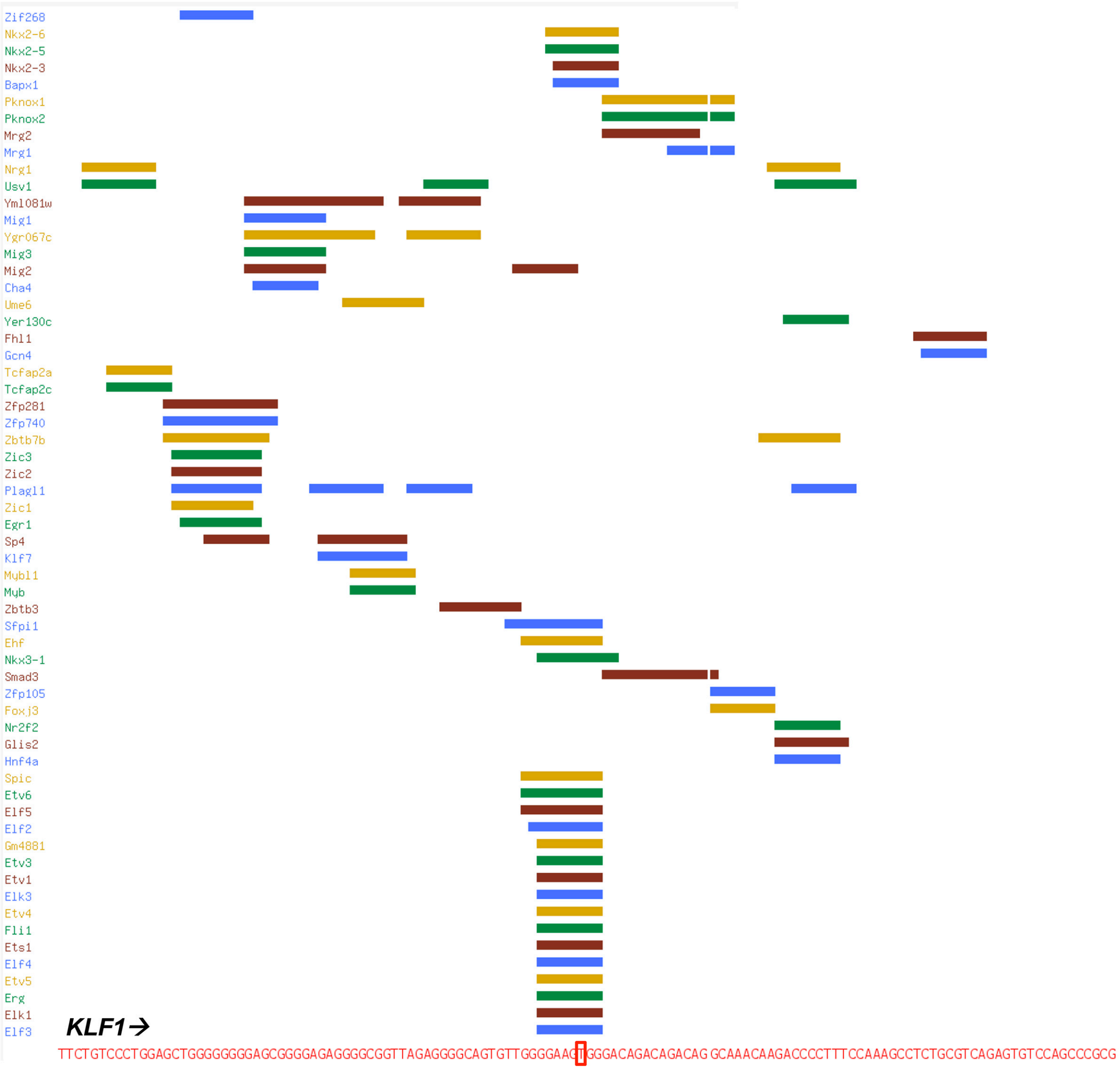

Figure S4. UniPROBE analysis of the region surrounding the intron 1 site.
100 nucleotide sequence surrounding the intron 1 site of interest (marked in red; oriented from left to right) was searched by the UniPROBE program [56,57] to identify potential cognate binding transcription factors. Among the factors predicted to recognize the intron 1 site are multiple members of the Ets family of proteins. The single base intron 1 mutation (T to C) is predicted to disrupt the binding for these proteins [57].

Supplemental Figure S5

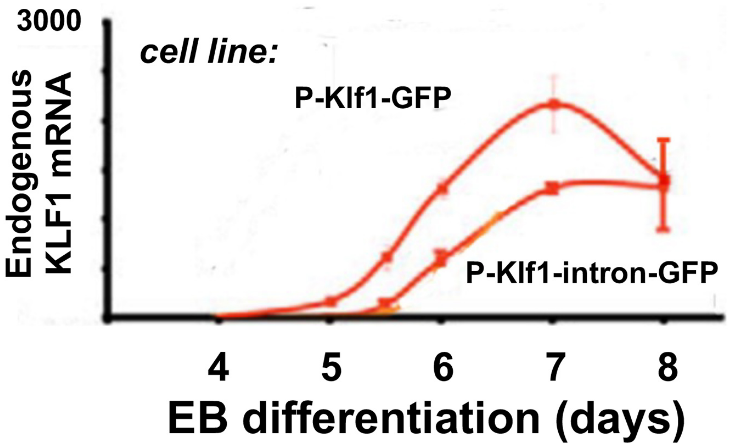

Figure S5. Expression of endogenous KLF1 expression in the differentiating ES cells used in Figure 1.

Reanalysis of data from a study [37] on the mouse *KLF1* promoter. Samples are the same as used in Figure 1a (from two stable ES lines ("P-Klf1-GFP" and "P-Klf1-intron-GFP"). These were quantitatively analyzed for expression of endogenous mouse KLF1. Data is from analysis of biological triplicates.

Supplemental Figure S6

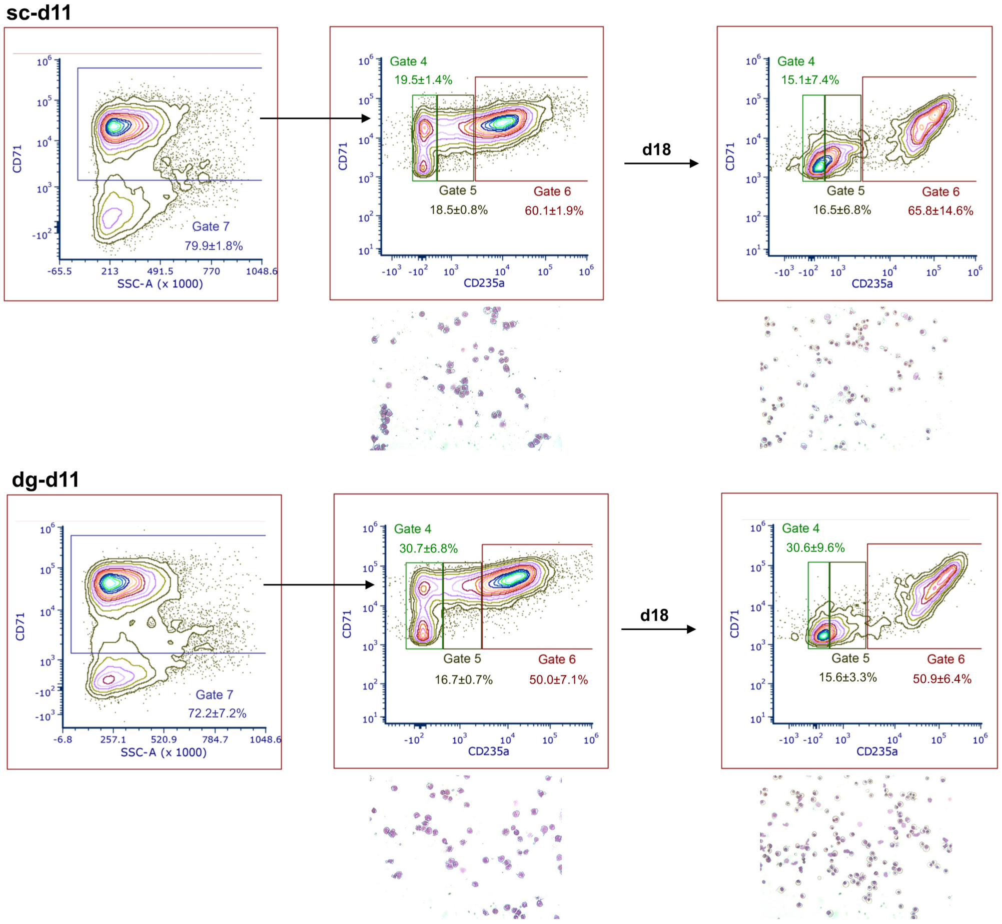

Figure S6. Expansion/differentiation analysis of CD34+ cells.

Human CD34+ cells that had been transfected with Cpf1 RNP containing scrambled gRNA ('sc') or Cpf1 RNP containing a dual guide mix of intron 1-directed gRNAs ('dg') were cultured and harvested at the end of phase II (day 11) or at the end of phase III (day 18) and analyzed for extent of differentiation by CD71 and CD235a cell surface marker expression. Representative FACS from analysis of biological triplicate samples are shown, along with ave±SD. Total CD71+ cells gated for CD235a -lo, -mid, or -hi are as indicated (gates 4,5,6, respectively). Overall patterns are similar across all samples and not significantly different (p=0.08-0.18). Inserts (below) show brightfield pictures of representative cytospins from the same samples for each time point/condition.

Supplemental Figure S7

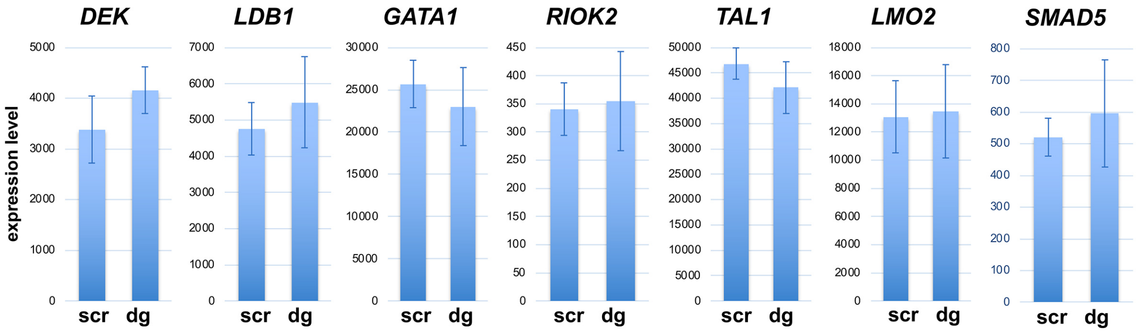

Figure S7. Expression of upstream KLF1 regulators.

RNA seq data from biological triplicate samples of control compared to intron 1-edited CD34+ cells was analyzed for expression of genes implicated in regulation of KLF1 expression. Control cells were transfected with control scrambled gRNA/RNP (scr) or intron 1-directed dual gRNA/RNP (dg). The source file for these genes is Table S7.

Supplemental Figure S8

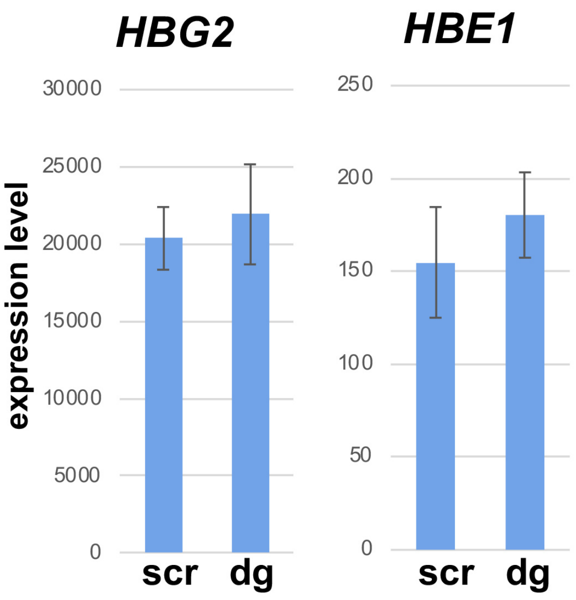

Figure S8. Expression of fetal and embryonic globin genes.
RNA seq data from biological triplicate samples of control compared to intron 1-edited CD34+ cells was analyzed for expression of γ- or ε-globin genes. Cells were transfected with control scrambled gRNA/RNP (scr) or intron 1-directed dual gRNA/RNP (dg). Repressors of γ-globin (*EIF2AK1, NFIX, ZNF410, ZBTB7A*, *PPP6C*, *PTPB1*, *NF1A*, *ATF4*, or *PUM1*) were not significantly changed in expression (not shown). The source file for these genes is Table S7.

Supplemental Figure S9

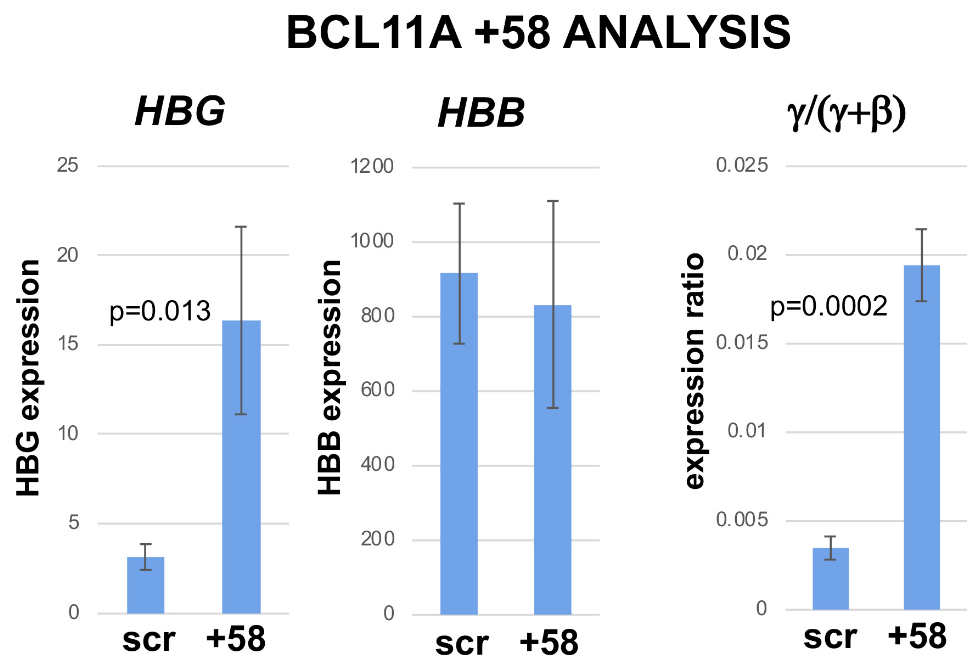

Figure S9. Globin expression after deletion of the +58 enhancer of the BCL11A gene. RNA seq data from biological triplicate samples of control compared to BCL11A/+58-edited CD34+ cells [66] was analyzed for expression of γ- or ß-globin genes by RT-qPCR; γ/(γ+β) ratios were calculated. Cells were transfected with control scrambled gRNA/RNP (scr) or BCL11A-directed gRNA/RNP (+58).
