# Supplementary material for: Identification of a genomic DNA sequence that quantitatively modulates KLF1 transcription factor expression in differentiating human hematopoietic cells
Source: Sci Rep. 2023 May 10;13:7589. doi: 10.1038/s41598-023-34805-5 (PMC10172341; doi:10.1038/s41598-023-34805-5)
Supplement: Supplementary file 2 — Supplementary Tables. [file 41598_2023_34805_MOESM2_ESM.pdf]

**Table S1. JMML patient samples**

| | UPN | sample ID | source | Hb F % | comments |
|---|---|---|---|---|---|
| 1 | 987 | HM1432 | BM | | |
| 2 | 1358 | HM2109 | PB | 42 | Homozygous -577 del, rs201870270, MAF=0.8% |
| 3 | 1441 | HM2176 | BM | | |
| 4 | 1484 | HM2230 | PB | | |
| 5 | 1603 | HM2429 | PB | | |
| 6 | 1669 | HM2534 | PB | | |
| 7 | 1716 | HM2620 | BM | | |
| 8 | 1905 | HM2936 | PB | | |
| 9 | 1911 | HM2948 | BM | 9.7 | |
| 10 | 1965 | HM3049 | BM | 19.7 | |
| 11 | 2024 | HM3152 | BM | | |
| 12 | 2048 | HM3200 | PB | | |
| 13 | 2065 | HM3235 | PB | | |
| 14 | 2099 | HM3294 | PB | 3.4 | Heterozygous -625 T>G mutation; non-conserved region |
| 15 | 2104 | HM3304 | PB | 57.1 | |
| 16 | 2114 | HM3314 | BM | 6.9 | |
| 17 | 2218 | HM3554 | PB | 4 | Heterozygous T>C, non-SNP mutation; intron 1 conserved region |
| 18 | 2292 | HM3662 | BM | 26 | |
| 19 | 2301 | HM3679 | BM | 20 | |
| 20 | 2309 | HM3690 | BM | 4.6 | |

## TABLE S2

**PROGNOS Summary for _home_apache_public_html_bao_Research_BioinformaticTools_prognos_03272019OHU99nls_Ranking-TALENv2.0**

The hg19 genome was searched for the TALEN pair targeting:

5'- TAGAGGGGCAGTGTTGG NN..NN GACAGACAGGCAAACAAG -3'

Using these RVDs:

Left: 01NI02NN03NI04NN05NN06NN07NN08HD09NI10NN11NG12NN13NG14NG15NN16NN

Right: 01NG02NG03NN04NG05NG06NG07NN08HD09HD10NG11NN12NG13HD14NG15NN16NG17HD

**Up to 0 mismatches were allowed in each nuclease half-site.**

Allowed spacing distances were: 10 11 12 13 14 15 16 17 18 19 20 21 22 23 24 25 26 27 28 29 30

Homodimers and heterodimers of the nucleases were included in the output

1 total sites were located

Mismatch Types Genomic Regions

0 and 0: 1

Exons: 0

Promoters: 0

Introns: 1

Intergenic: 0

The top 1 sites are ranked below according to parameters optimized for TALEN off-target prediction.

Rank: 1 Homology Score: 2 TALEN Score: 100

Genomic Region: Intron Closest Gene: KLF1

**Up to 3 mismatches were allowed in each nuclease half-site.**

Allowed spacing distances were: 10 11 12 13 14 15 16 17 18 19 20 21 22 23 24 25 26 27 28 29 30

Homodimers and heterodimers of the nucleases were included in the output

1 total sites were located

Mismatch Types Genomic Regions

0 and 0: 1

Exons: 0

Promoters: 0

Introns: 1

Intergenic: 0

The top 1 sites are ranked below according to parameters optimized for TALEN off-target prediction.

Rank: 1 Homology Score: 32 TALEN Score: 100

Genomic Region: Intron Closest Gene: KLF1

**TABLE S3. CRISPOR analysis of gRNA designs**

| | |
|---|---|
| Genome | hg38 |
| PAM | TTTV |
| Version | CRISPOR 4.99 |

| guideId | TargetSeq | Off-targets for 0-1-2-3-4 mismatches | offtargetCount | targetGenomeGeneLocus | DeepCpf1-Score |
|---|---|---|---|---|---|
| 28forw | *TTTC* TGTCCCTGGAGCTGGGGGGGG | 0-0-0-2-30 | 32 | intron:KLF1 | 7.54 |
| 110rev | *TTTG* CCTGTCTGTCTGTCCCACTTC | 0-0-0-7-78 | 85 | intron:KLF1 | 28.59 |

**Table S4. Sequence information**

TALEN primer pair
Talen-L
TAGAGGGGCAGTGTTGG
Talen-R
GACAGACAGGCAAACAAG

Coding sequences for gRNAs [PAM in brackets]
Cpf1-a
[TTTC] TGTCCCTGGAGCTGGGGGGGAG
Cpf1-b
GGGAAGTGGGACAGACAGACAGG [CAAA]
scr (negative control)
CGTTAATCGCGTATAATACGG

Deep sequencing genomic primer pair
MGH-L
CCTGCCAGTTTTCCATCTCC
MGH-R
CTGACGCAGAGGCTTTGGAA

RNA-qPCR primer pair
hKLF1-F
TTGCGGCAAGAGCTACACCAAG
hKLF1-R
GTAGTGGCGGGTCAGCTCGTC

ETV6 ChIP primers
INT1chipF1
TGATCGGTTTCTGTCCCTGG
INT1chipR1
AAGGGGTCTTGTTTGCCTGT

INT1chipF2
CCGAGTGTGGTTCCAGATAGT
INT1chipR2
TGTCTGTCCCACTTCCCCAA

ETV6 positive control primers from J Pimanda
HHEX+1For
CCTGACCCTTTCCGTTCATA
HHEX+1Rev
ATGCAGCCAGGAAAACACTT

GATA2+3.5For
TTACGCATTATTTGCAGAGTGGAG
GATA2+3.5Rev
GCAGCCTGAAGATAAGGAAACTTC

LMO2-25For
AGTGTGGTCTGGTGGTTAGCATAC
LMO2-25Rev
TGCGTTTCCTTCCCTGGTTGAG

<u>Globin primers for RT-qPCR</u>
hHBB-F
ACACAACTGTGTTCACTAGCAACCTCA
hHBB-R
GGTTGCCCATAACAGCATCAGGAGT

hHBA-F
AAGACCTACTTCCCGCACTTC
hHBA-R
GTTGGGCATGTCGTCCAC

hHBG-F
TGGATGATCTCAAGGGCAC
hHBG-R
TCAGTGGTATCTGGAGGACA

hHBE-F
AAACCTGTCGTCTCCCTCTG
hHBE-R
GTGAACTCCTTGCCAAAGTG

**Table S8. Bioinformatic links**

R
R Core Team (2022). R: A language and environment for sta0s0cal compu0ng. R Founda0on for Sta0s0cal Compu0ng, Vienna, Austria. URL h>ps://www.R-project.org/.

EnsDb.Hsapiens.v86
Rainer J (2017). EnsDb.Hsapiens.v86: Ensembl based annota0on package. R package version 2.99.0.

ggplot2
Wickham H (2016). ggplot2: Elegant Graphics for Data Analysis. Springer-Verlag New York. ISBN 978-3-319-24277-4, h>ps://ggplot2.0dyverse.org.

readr
Hadley Wickham, Jim Hester and Jennifer Bryan (2022). readr: Read Rectangular Text Data. R package version 2.1.2. https://CRAN.R-project.org/package=readr

ggrepel
Kamil Slowikowski (2021). ggrepel: Automatically Position Non-Overlapping Text Labels with 'ggplot2'. R package version 0.9.1. https://CRAN.R-project.org/package=ggrepel

dplyr
Hadley Wickham, Romain François, Lionel Henry and Kirill Müller (2022). dplyr: A Grammar of Data Manipulation. R package version 1.0.9. https://CRAN.R-project.org/package=dplyr

EnhancedVolcano
Blighe K, Rana S, Lewis M (2022). EnhancedVolcano: Publication-ready volcano plots with enhanced colouring and labeling. R package version 1.14.0, https://github.com/kevinblighe/Enhanc
